# QTL associated with resistance to *Stagonosporopsis citrulli* in *Citrullus amarus*

**DOI:** 10.1038/s41598-022-23704-w

**Published:** 2022-11-15

**Authors:** Lincoln Adams, Cecilia McGregor

**Affiliations:** Institute of Plant Breeding, Genetics, and Genomics, University of Georgia, Athens, GA USA

**Keywords:** Genetics, Molecular biology, Plant sciences

## Abstract

Gummy stem blight (GSB) is a fungal disease affecting cucurbit crops, including watermelon (*Citrullus lanatus*), leading to significant yield losses. The disease is caused by three *Stagonosporopsis* species, of which *Stagonosporopsis citrulli* is the most common in the southeastern United States. Currently no gummy stem blight-resistant watermelon cultivars are available to growers. In this study, QTL-seq in an interspecific population developed from Sugar Baby × PI 189225 (*Citrullus amarus*) identified QTL on chromosomes 2, 5, 9 and 11. A novel QTL on chromosome 5 (*Qgsb5.2*) associated with resistance to *S. citrulli* (PVE = 13.3%) was confirmed by genetic mapping. KASP marker assays were developed for selection of *Qgsb5.2* to allow breeders to track the allele contributing resistance to GSB, reducing the need for laborious phenotyping. Pyramiding different GSB resistance QTL could be a useful strategy to develop GSB resistant watermelon cultivars.

## Introduction

Gummy stem blight (GSB) is a fungal disease which affects cucurbit crops globally, leading to decreases in yield and revenue loss for growers^1,2^. Watermelon (*C. lanatus*) is one of the most severely affected crops with average yield losses of up to 43% in unsprayed plots^3^. Until recently it was believed that GSB was caused by a single pathogen, *Didymella bryoniae*. However, Stewart et al. ^2^ determined that GSB is caused by three distinct species of *Stagonosporopsis*, *S. cucurbitacearum, S. caricae* and *S. citrulli*^2^. Currently, GSB is controlled using costly fungicides that are damaging to the environment highlighting the need for resistant cultivars^4,5^.

Initial efforts to breed watermelon with GSB resistance were conducted working under the hypothesis that the resistance in PI 189225 (*C. amarus*) was controlled by a single gene, designated *db*^6^. Norton used two resistant accessions, PI 189225 and PI 271778 (*C. lanatus*), to develop four cultivars (‘AU-Producer’, ‘AU-Jubilant’, ‘AU-Sweet Scarlet’, and ‘AU-Golden Producer’) with reported GSB resistance^7–9^. Unfortunately, these varieties did not exhibit the same level of GSB resistance once they were grown by producers on a larger scale^10^. Additional sources of GSB-resistant watermelon germplasm have been identified by other researchers in recent years^11–13^; however, to date, none of these discoveries have led to the release of GSB-resistant commercial watermelon cultivars. Recently, Gusmini et al.^14^ demonstrated that GSB resistance in four crosses between elite watermelon cultivars and resistant PIs, including PI 198225, is quantitatively inherited^14^.

In three previous studies, researchers identified GSB resistance QTL in watermelon. Ren et al*.*^15^ used a population derived from K3 (*C. lanatus*) × PI 189225 and an isolate of *S. cucurbitacearum* to map a GSB resistance QTL on chromosome 8^15^. Lee et al*.*^16^ used a population derived from 920533 (*C. lanatus*) × PI 189225 and “*D. bryoniae* ‘KACC 40937 isolate’” to map GSB resistance QTL, two on chromosome 8 and one on chromosome 6^16^. The QTL mapped by the two studies on chromosome 8 are distinct from one another. The species of *Stagonosporopsis* used by Lee et al.^16^ is unknown. Gimode et al.^17^ used a population derived from Crimson Sweet (*C. lanatus*) × PI 482276 (*C. amarus*) and *S. citrulli* isolate 12178A to map GSB resistance QTL on chromosomes 5 and 7^17^. Differences in the locations of these QTL, especially those derived from the same resistant parent (PI 189225), are likely due to either differences in resistance to the various species or isolates of *Stagonosporopsis* used for phenotyping or differences in phenotyping methodologies. Lee et al*.*^16^ evaluated lesion severity on the stems separately from leaf lesions, while Ren et al.^15^ scored only leaf lesions and Gimode et al*.*^17^ scored the entire seedling^15–17^.

QTL-seq, a modification of bulked segregant analysis, was proposed by Takagi et al.^18^ as a quick and relatively cheap method to identify QTL associated with a particular trait^18,19^. This technique has been widely used to identify QTL for various traits, including blast resistance in rice, late spot resistance in peanut, and Fusarium wilt and GSB resistance in watermelon^15–18,20–22^.

The aim of the current study was to use QTL-seq to identify loci associated with resistance to *S. citrulli,* the most common GSB-causing *Stagonosporopsis* species in the southeastern US, in a population derived from a cross between Sugar Baby (SB, susceptible) and PI 189225 (resistant) and to develop KASP marker assays for selection of resistance loci in watermelon^2,23^.

## Results

### Phenotypic data

**Figure 1:**
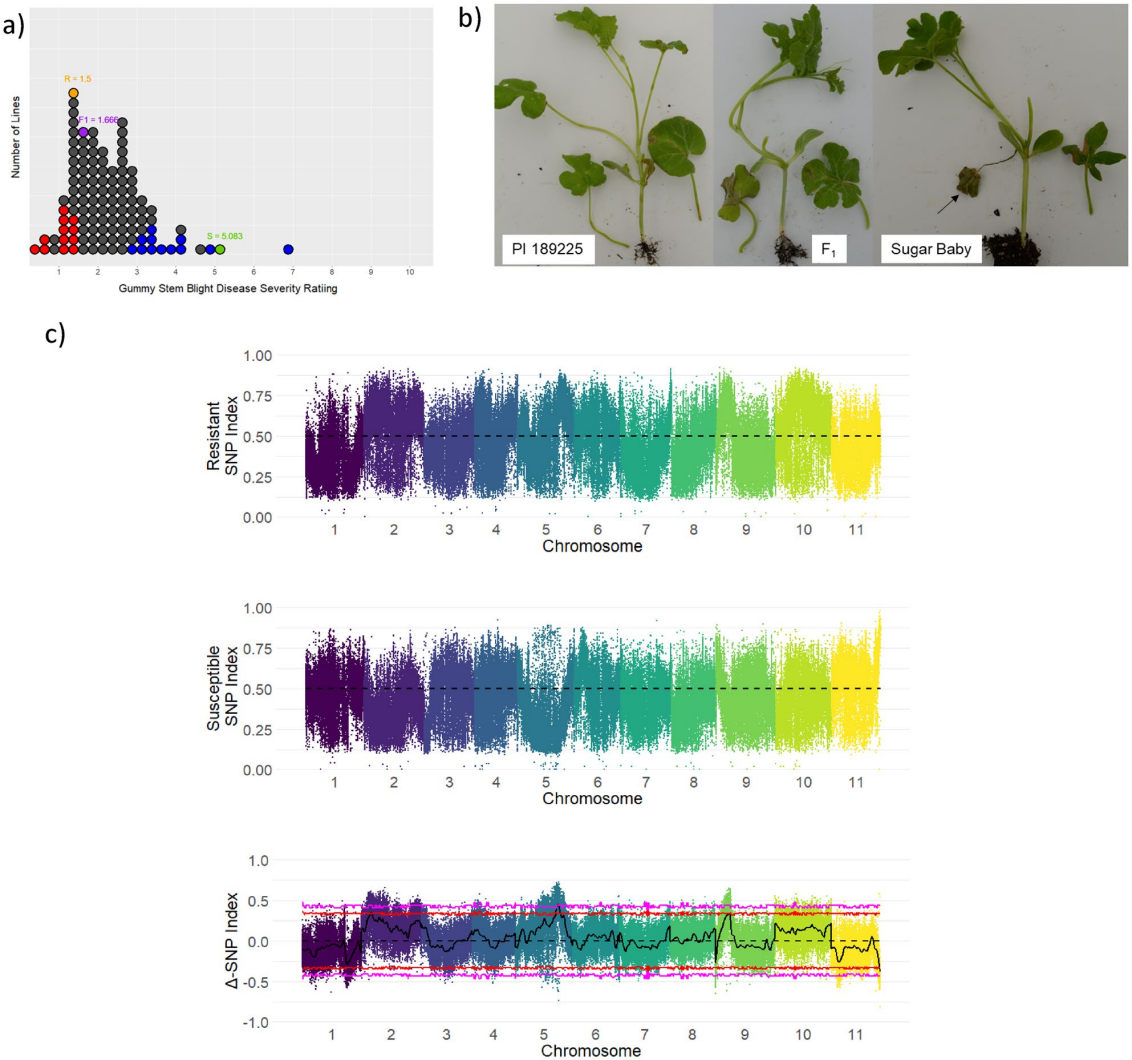
(**a**) Stacked dot plots showing the distribution of the average disease severity of the F_2:3_ families (n = 111) of a cross between Sugar Baby (S) and PI 189225 (R). Red and blue dots represent the families selected to generate the resistant and susceptible DNA bulks, respectively. The orange, purple, and green dots represent the average phenotypic values for PI 189225, the F_1_, and Sugar Baby, respectively. (**b**) Gummy stem blight disease severity in PI 189225 (resistant), the F_1,_ and Sugar Baby (susceptible) after infection with *S. citrulli*. The black arrow indicates a completely dead leaf in the susceptible parent. (**c**) The SNP index for the gummy stem blight resistant and susceptible bulks and the Δ-SNP index of each identified SNP. Individual colored points represent individual SNPs, with each color representing a different chromosome. The black line represents the tricube smoothed Δ-SNP index value using a 1 Mb sliding window. The red line represents the 95% confidence interval and the pink line represent the 99% confidence interval. A positive Δ-SNP index represents SNPs differing from the reference genome which are potentially contributing to the trait of interest, while regions with a negative Δ-SNP index represent SNPs shared with the reference genome which are potentially contributing to the trait of interest. Regions where the black line exits the bounds of the confidence intervals represent potential QTL for gummy stem blight resistance. Δ-SNP indices were calculated using QTLseqr^48^.

The phenotypic distribution for the population was skewed (Shapiro–Wilk test *P* =  < 0.0001) towards lower disease symptom severity (Fig. 1a). PI 189225 (resistant parent), Sugar Baby (susceptible parent) and the F_1_ had disease severity scores of 1.5, 4.6 and 1.7, respectively (Fig. 1b). Broad sense heritability for disease severity was 0.27. The 20 most resistant and 20 most susceptible families from the first screen were re-screened (data not shown) and the 12 most resistant and susceptible plants across the two screens were used to create the resistant and susceptible bulks for QTL-seq. The average disease severity scores of the resistant and susceptible bulks over the two screens were 1.5 and 3.7, respectively.

### QTL-seq

DNA sequences were obtained from each bulk consisting of 148,037,545 reads for the susceptible bulk and 149,620,933 reads for the resistant bulk. Alignment to the *C. lanatus* 97103_v2 genome resulted in 145,179,598 mapped reads with a mapping ratio of 98.07%, with 89.53% of reads properly paired and an average coverage of 56 × for the susceptible bulk, and 147,148,116 mapped reads with a mapping ratio of 98.53% with 89.79% of reads properly paired and an average coverage of 57 × for the resistant bulk^24^. 6,186,663 SNPs were identified from the aligned reads (Table 1). Initial filtering with GATK reduced the number of SNPs to 5,806,149. The second round of filtering using QTLseqr filtered another 5,567,799 leaving 238,350 high quality SNPs for analysis. Δ-SNP Index analysis using QTLseqr revealed four regions in which the tricube smoothed Δ-SNP Index exceeded the 95% confidence threshold, one region each on chromosomes 2, 5, 9, and 11 (Fig. 1c, Table 2). The peak region on chromosome 2 exceeding the 95% confidence interval spanned 1,037,352 base pairs, from 5,835,675 to 6,873,027 bp, with a peak Δ-SNP index of 0.36. The peak region on chromosome 5 exceeding the confidence interval spanned 2,904,205 base pairs, from 25,465,570 to 28,369,775 bp, with a peak Δ-SNP Index of 0.45. The peak on chromosome 5 exceeded the 99% confidence interval. The peak region on chromosome 9 exceeding the 95% confidence interval spanned 379,167 base pairs, from 8,570,733 to 8,949,900 bp, with a peak Δ-SNP Index of 0.34. The peak region on chromosome 11 exceeding the 95% confidence interval spanned 503,798 base pairs, from 30,375,412 to 30,879,210 bp, with a peak Δ-SNP Index of − 0.38.

**Table 1:** Mapping statistics for short reads generated by sequencing of the resistant and susceptible DNA bulks.

Sample	Total reads	Mapped reads	Mapping ratio (%)	Properly paired (%)	Average coverage (x)
Susceptible bulk	148,037,545	145,179,598	98.07%	89.53%	56
Resistant bulk	149,620,933	147,148,116	98.35%	89.79%	57

**Table 2:** Quantitative trait loci identified for gummy stem blight resistance by comparing the susceptible and resistant bulks of the Sugar Baby × PI 189225 F_2:3_ population. Locations are based on the *C. lanatus* 91703_v2 genome sequence (Guo et al*.*^24^).

Chr	Start (bp)	End (bp)	Length (bp)	Total # of SNPs	Peak Δ-SNP index	Peak Δ-SNP index position
2	5,835,675	6,873,027	1,037,352	482	0.36	6,517,652
5	25,465,570	28,369,775	2,904,205	1677	0.45	26,916,783
9	8,570,733	8,949,900	379,167	174	0.34	8,843,908
11	30,375,412	30,879,210	503,798	147	− 0.38	30,879,210

### QTL mapping

**Figure 2:**
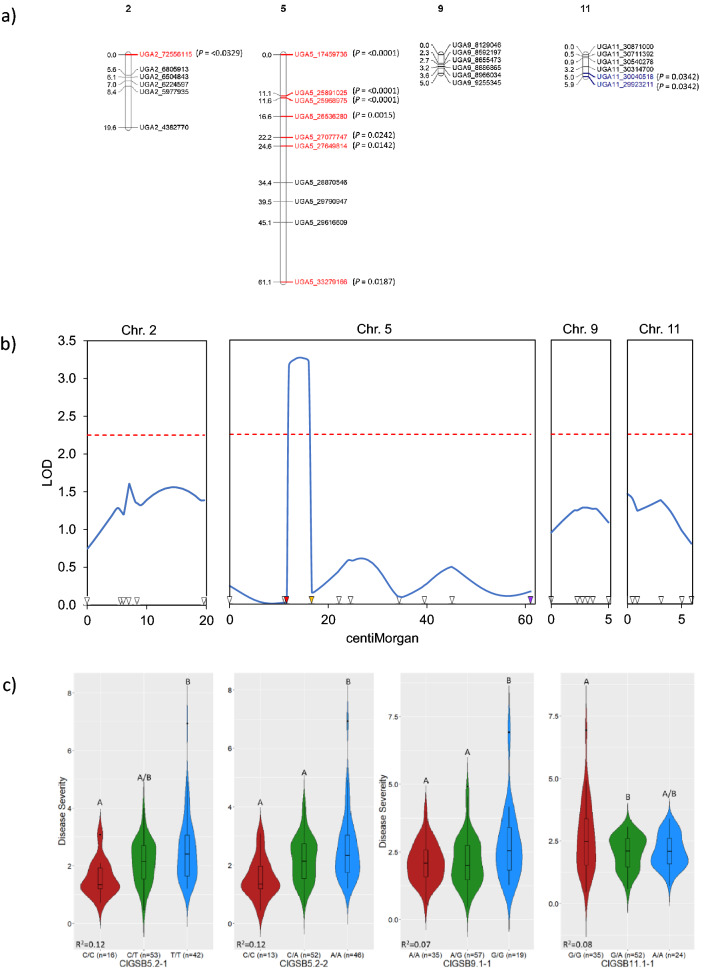
(**a**) Linkage maps of the regions on chromosomes 2, 5, 9, and 11 identified by QTLseq. These maps are comprised of 6, 10, 6 and 6 markers respectively with lengths of 19.6, 61.1 5.0 and 5.9 cM. Markers indicated in red are preferentially segregating for the alleles contributed by SB, and markers indicated in blue are preferentially segregating for the alleles contributed by PI 189225. *P* values for Chi^2^ goodness of fit test are given next to each marker. (**b**) QTL associated with Gummy stem blight resistance in the Sugar Baby × PI 189225 F_2:3_ population (n = 111). The red dashed line indicates the LOD threshold (1000 permutations), while the triangles indicate the position of the KASP markers. The purple triangle represents marker ClGSB5.1-1, which was linked to *Qgsb5.1* in the Gimode et al*.* study^17^. The red triangle represents marker ClGSB5.2-1, and the orange triangle represents marker ClGSB5.2-2, both of which are linked to *Qgsb5.2*. (**c**) Violin plots showing the association of ClGSB5.2-2, ClGSB5.2-2, ClGSB9.1-1 and ClGSB11.1-1 genotypes with disease severity in the Sugar Baby × PI 189225 population (n = 111). Red plots represent individuals that are homozygous for the allele from the resistant parent (PI 189225) allele, while blue plots represent individuals homozygous for the susceptible parent (Sugar Baby) allele. Box and whisker plots show median and quartile ranges of each group. Different letters above the plots indicate significant differences (*P* < 0.05) based on a Tukey–Kramer test.

Twenty-eight KASP marker assays were developed spanning the chromosomal regions of interest. Genetic mapping resulted in four linkage groups spanning 19.61, 61.06, 5.01, and 5.90 cM, on chromosomes 2, 5, 9, and 11, respectively (Fig. 2a). Segregation distortion was observed for several markers on the map, predominantly in the direction of the susceptible SB parent (Fig. 2a). QTL mapping (Fig. 2b) resulted in a single significant peak on chromosome 5, between SNP UGA5_25968975 (KASP assay ClGSB5.2–1) and UGA5_26536280 (KASP assay ClGSB5.2–2) at 14.0 cM (LOD = 3.37; PVE = 13.3%), with left and right 1-LOD confidence intervals of 11.5 cM and 16.5 cM. Additive and dominance values for this QTL were 0.5414 and 0.1116, respectively. Significant preferential segregation of the susceptible alleles was observed for seven out of the ten markers mapped on chromosome five (χ^2^ *P* < 0.05). To determine whether the QTL identified in the current study overlaps with *Qgsb5.1* (syn*. ClGSB5.1*) identified by Gimode et al*.*^17^, the marker most closely associated to *Qgsb5.1* (ClGSB5.1–1) was included in the map (Fig. 2b)^17^. ClGSB5.1–1 mapped 44.5 cM outside the confidence interval of the current QTL and it was therefore concluded that the QTL identified in the current study represents a novel QTL (*Qgsb5.2)*. For the two KASP assays closest to *Qgsb5.2*, individuals homozygous for the allele from the susceptible parent (A/A or T/T) were significantly less resistant to GSB than individuals homozygous for the allele from the resistant parent (C/C) (Fig. 2c, Table 3). Assay ClGSB5.2–1 showed significant (*P* = 0.001) association with disease resistance (C/C = 2.1; T/T = 2.9), with an R^2^ value of 12.1%. Assay ClGSB5.2–2 showed significant (*P* = 0.001) association with disease resistance (C/C = 1.6; A/A = 2.6), with an R^2^ value of 12.3%. The haplotype representing both ClGSB5.2–1 and ClGSB5.2–2 showed a significant (*P* = 0.014) association with disease resistance (C/C:C/C = 1.6; T/T:A/A = 2.6) for individuals homozygous at both loci for the resistant or susceptible alleles, with and R^2^ value of 12.2% (data not shown). The QTL identified by QTL-seq on chromosomes 2, 9, and 11 could not be confirmed by genetic mapping. However, single marker analysis found that individuals homozygous for the SB allele had significantly higher disease severity (*P* < 0.05) than heterozygotes or individuals homozygote for the PI 189225 alleles for all markers on chr. 9. On chr. 11, heterozygote individual had significantly lower disease severity than individuals homozygous for the PI 189225 alleles for four markers (UGA11_30314700, UGA11_30540278, UGA11_30711392 and UGA11_30871000). Individuals homozygous for the Sugar Baby allele, were not significantly different from heterozygotes or PI 189225 homozygotes. UGA9_8886865 (KASP assay ClGSB9.1; *P* < 0.0106; R^2^ = 8.5%; AA = 2.1; AG = 2.2; GG = 2.9) and UGA11_30871000 (KASP assay ClGSB11.1–1; *P* < 0.0203; R^2^ = 7.5%; GG = 2.7; GA = 2.18; AA = 2.1) were the most highly significant marker on chrs 9 and 11, respectively (Fig. 2c). For the markers on chr. 11, heterozygous individuals had lower disease severity than the individual homozygous for the allele from the resistant parent.

**Table 3:** KASP assays for flanking SNPs for GSB resistance QTL on chromosome 5 (*Qgsb5.2*).

KASP Assay	Ta (°C)	SNP Position	Primer type	Primer sequence (5′–3′)	Allele
ClGSB5.2–1	57	UGA5_25968975	FAM	GAAGGTGACCAAGTTCATGCT CCATTGGAATTGCCACTAGTTTGC	PI 189225 (R)
			VIC	GAAGGTCGGAGTCAACGGATTCCATTGGAATTGCCACTAGTTTGT	SB (S)
			Reverse	TCAAAGGTCTGCTGGCTCCT	
ClGSB5.2–2	57	UGA5_26536280	FAM	GAAGGTCGGAGTCAACGGATTCCTACTTTCCATGCACATGCTCTC	PI 189225
			VIC	GAAGGTGACCAAGTTCATGCTCCTACTTTCCATGCACATGCTCTA	SB
			Reverse	CCGGGTAACTGTCCAGATCG	
ClGSB9.1–1	55	UGA9_8886865	FAM	GAAGGTCGGAGTCAACGGATT AATGCATCAATATCAAGCCAAATCTAA	PI 189225
			VIC	GAAGGTGACCAAGTTCATGCT AATGCATCAATATCAAGCCAAATCTAG	SB
			Reverse	GTGAGATATTGTTTCTATGTGGAGCTAATA	
ClGSB11.1–1	55	UGA11_30871000	FAM	GAAGGTCGGAGTCAACGGATT GTATGCCCTCTTAATTTCGAGATTCTG	PI 189225
			VIC	GAAGGTGACCAAGTTCATGCT TGTATGCCCTCTTAATTTCGAGATTCTA	SB
			Reverse	ATTCTTTGTCCGTTTTAGGTTGTACATT	

### Synteny analysis and candidate genes

The 567,305 bp region of *Qgsb5.2* contained 38 predicted genes. Three of these genes, an Ethylene-responsive transcription factor 2 gene (*Cla97C05G096890*), a histidine kinase 5 gene (*Cla97C05G096980*) and an enhanced disease resistance 2-like protein gene (*Cla97C05G097030*), are probable fungal disease resistance genes based on their similarity to genes known to be related to fungal resistance in *Arabidopsis thaliana*^24–27^*.* No non-synonymous polymorphisms were detected in the exons of these genes within our sequencing data. The *Qgsb5.2* region was not found to be syntenic to any previously identified GSB resistance QTL in cucurbits^15–17,28–30^.

## Discussion

GSB causes significant yield losses for watermelon growers^3^. Efforts to control the disease using genetic resistance have recently been confounded by the discovery that the disease is caused by three *Stagonosporopsis* species^2^. A study by Gimode et al.^31^ showed significant differences in disease severity caused by different *Stagonosporopsis* isolates in different watermelon genotypes, but no significant genotype × isolate interactions were observed^31^. Unraveling the host resistance response to different *Stagonosporopsis* species and isolates is likely key to developing cultivars with field resistance to this disease. Here we report a novel QTL from PI 189225 associated with resistance to *S. citrulli*, the most common species causing GSB in the southeastern US.

A continuous distribution was observed for disease symptom severity in the Sugar Baby × PI 189225 population, indicating quantitative control of GSB resistance in this population. Additionally, both resistant and susceptible families of the population exhibited transgressive segregation relative to the parents. This contributes additional evidence to the hypothesis that GSB resistance is quantitatively controlled^14^. Other recent studies have reached similar conclusions, in contrast to earlier reports which proposed resistance in PI 189225 was monogenic^6,14,15,17,28–30,32–34^.

QTL-seq identified QTL on chromosomes 2, 5, 9, and 11, and *Qgsb5.2* was confirmed by genetic mapping. Similar results were obtained by Ramos et al*.*^35^ in a study mapping *Phytophthora capsici* resistance in squash^35^*.* Significant single marker associate was also found with markers on chromosomes 9 and 11, but not on chromosome 2. Possible explanations for the inability to confirm certain QTL, include the small population size and/or a small contribution to resistance by individual QTL^36^. The unconfirmed QTL could also have been a false positive.

The KASP assays (ClGSB5.2–1, ClGSB5.2–2, ClGSB9.1–1 and CLGSB11.1–1) were effective at explaining between 7 and 12% of the resistance observed in this population. While this shows that the markers can effectively track the loci, it also underlines the need for the identification of more resistance QTL, and to pyramid different QTL as a 12% increase in resistance would likely not lead to any appreciable reduction in fungicide use by farmers. The two markers most closely linked to *Qgsb5.2* (ClGSB5.2–1 and ClGSB5.2–2) exhibited severe segregation distortion (ClGSB5.2–1: *P* < 0.0001; ClGSB5.2–2: *P* = 0.0015) in the direction of the SB alleles. Segregation distortion was observed for all markers within 12 cM of *Qgsb5.2*. The significant segregation distortion is likely due to the interspecific nature of the cross which originated this population. Segregation distortion in *C. lanatus* × *C. amarus* crosses is a common phenomenon (Sandlin et al*.* 2012, Gimode et al*.*^17^) and complicates introgression of desirable alleles from *C. amarus* into elite watermelon.

Two previous studies using the same resistant source (PI 189225) identified two different QTL on chromosome 8 and one on chromosome 6^15,16^. A possible explanation for the different QTL identified in different studies is the use of different phenotyping methods or rating systems. The study by Lee et al.^16^ rated stem lesions and leaf lesions separately and mapped both separately, while Ren et al.^15^ rated plants based on the symptoms on four true leaves^15,16^. The current study rated plants based on the symptoms of the first two true leaves. Another possible explanation for the identification of different QTL is the use of different species of the GSB-causing fungi. Ren et al*.*^15^ used an isolate of *S. cucurbitacearum,* while the current study was conducted using an isolate of *S. citrulli*^15^. The specific species of *Stagonosporopsis* used by Lee et al*.*^16^ is not known. These three studies suggest that different loci might control resistance to different species of *Stagonosporopsis* causing GSB. Ideally, repeated studies using the same phenotyping conditions and rating systems should be conducted under field conditions, where evidence is emerging that populations of multiple species of *Stagonosporopsis* can exist within a single field^37^.

*Qgsb5.2* is at a different location than the QTL identified by Gimode et al.^17^ using the same *S. citrulli* isolate (12178A), but a different resistant parent (PI 482276). This seems to indicate that the two PIs contain distinct resistance loci. This could be beneficial to breeders as it would allow their effects to be combined through gene pyramiding.

Of the 38 predicted genes within the *Qgsb5.2* region, there are three genes of particular interest for their potential association with disease resistance. *Cla97C05G096980* is homologous to the histidine kinase 5 gene of *A. thaliana*, which has been shown to regulate the production of reactive oxygen species in response to stressors including necrotrophic fungi^25^. *Cla97C05G097030* is similar to the *enhanced disease resistance 2* gene in *A. thaliana* which has been shown to negatively regulate salicylic acid-based defenses and cell death in powdery mildew infections^27^. *Cla97C05G096890* is homologous to the *Ethylene-responsive transcription factor 2* gene from *A. thaliana*, which has been shown to be related to signaling pathways involved in resistance to *Alternaria brassicicola*^38^. Interestingly, salicylic acid-based signaling tends to be related to defense against biotrophic disease such as powdery mildew, while ethylene based signaling tends to be related to defense against necrotrophic diseases like those caused by *A. brassicicola*^39^. Given that GSB causing *Stagonosporopsis* species are thought to feed necrotrophically, *Cla97C05G096980* and *Cla97C05G096890* are likely candidate genes for this QTL. Further gene expression and gene knockout studies are needed to confirm the potential role of these genes in GSB resistance.

The current study used the *C. lanatus* 97103_v2 genome^24^ for analysis, since at the time, the *C. amarus* genome was not publicly available. However, additional resistance genes might be present in *C. amarus* that could be missed in the present study. It will be interesting to investigate these regions of interest further using the *C. amarus* genome and the watermelon Pan-genome that is currently in development (Shan Wu, personal communication).

The identification of *Qgsb5.2* brings the total number of QTL identified for GSB resistance to five. However, it remains unclear if these QTL contribute resistance to all GSB causing *Stagonosporopsis* species and isolates. More research is needed to determine the utility of these QTL against different species and isolates of *Stagonosporopsis* under field conditions. However, in light of the discovery of distinct QTL by this study, Ren et al.^15^, and Lee et al.^17^ in germplasm derived from the same resistant PI, it is beginning to appear as if field level GSB resistance will require the incorporation of a number of different QTL^15,16^. Pyramiding multiple resistance genes can allow breeders to develop plants that are resistant to many different isolates or species of disease-causing pathogens, while increasing the durability of resistance to individual pathogen isolates, as shown for wheat stem rust^40^. QTL mapping resistance to multiple isolates of GSB-causing fungi in the same study would contribute greatly to our understanding of this issue and may provide the requisite QTL for pyramiding. The QTL identified in this study represents one part of that potential resistance gene pyramid to reduce the need to spray fungicides in order to control the disease.

## Materials and methods

### Plant materials

A cross was made between Sugar Baby (*C. lanatus*, susceptible) and PI 189225 (*C. amarus*, resistant) and a single plant from the resulting F_1_ was selfed to generate F_2_ seed. F_2_ plants were selfed in the greenhouse to produce 111 F_2:3_ families. Leaf material collected from the parents, F_1_ and F_2_ plants were stored at − 80 °C until use.

### Inoculum preparation

*Stagonosporopsis citrulli* isolate 12178A (provided by M. Brewer, collected in Berrien County, Georgia, U.S.A in 2012) was grown on full strength potato dextrose agar (PDA) for four weeks and then subcultured on quarter-strength PDA for a further 17 days. Conidia was harvested by flooding each Petri dish with 10 mL 0.1% Tween-20 (MilliporeSigma, St. Louis, MO) and scraping the plate with a microscope slide. The solution was filtered through 3 layers of cheese cloth and conidia were quantified under a microscope using a hemocytometer (Hausser Scientific, Horsham, PA). The conidial solution was diluted to 500,000 conidia per mL prior to inoculation. Due to low yields, a concentration of 420,000 conidia per mL was used for the re-screen.

### Resistance screening

Initial resistance screening was conducted between March 20th and April 8th, 2019, in a greenhouse in Athens, GA. Seed from the parents, F_1_ and 111 F_2:3_ families were sown in 48-well seedling trays in a randomized complete block design with four plants per block and four blocks. Two additional trays containing controls and 12 of the F_2:3_ families were also sown for mock inoculations. Seedlings were grown in a greenhouse with supplemental lighting until the 2–3 true leaf stage (16 days) and inoculated by spraying inoculum on the seedlings with a hand spray bottle until run-off. The mock inoculations were sprayed with 0.1% Tween-20 solution. After inoculation, the plants were placed inside a plastic tunnel with two humidifiers (Trion IAQ, Sanford, NC) in the greenhouse (average temperature = 24.0 °C, average humidity = 98.8%). After 72 h, the seedlings were moved to a greenhouse bench and overhead watered twice per day for four days. At 7 days post inoculation, the seedlings were rated for disease severity using a 0–10 scale (0: no lesions present on the first two true leaves, 1: 1–10% of first two true leaves covered in lesions, 2: 11–20% of first two true leaves covered in lesions, 3: 21–30% of first two true leaves covered in lesions, 4: 31–40% of first two true leaves covered in lesions, one leaf beginning to collapse, 5: 40–50% of first two true leaves covered in lesions, 1 true leaf collapsed, 6: 1 of first two true leaves dead, other 1–25% of true leaves covered in lesions, 7: 1 of first two true leaves dead, other 26–50% covered in lesions, 8: 1 of first two true leaves dead, other 51–75% covered in lesions, 9: both first two true leaves dead, 10: seedling totally collapsed or dead). The mean severity rating for each family/control was calculated using JMP® version 15.0.0 (SAS Institute Inc., Cary, NC). A Shapiro–Wilk test was conducted using JMP to test the normality of the phenotypic distribution. Broad sense heritability (H^2^) was calculated as described by Kearsey and Pooni^41^: $$H^{2} = \frac{{V_{{F_{2} }} - {\raise0.7ex\hbox{$1$} \!\mathord{\left/ {\vphantom {1 4}}\right.\kern-\nulldelimiterspace} \!\lower0.7ex\hbox{$4$}}\left( {V_{{P_{1} }} + V_{{P_{2} }} + 2V_{{F_{1} }} } \right)}}{{V_{{F_{2} }} }},$$ where V_P1_, V_P2_, V_F1_ and V_F2_ represents the variation among plants from parent 1, parent 2, the F_1_ and F_2_, respectively.

The 20 most resistant and 20 most susceptible families were re-screened using the same protocol between May 1st and May 25th, 2019 (average temperature = 23.4 °C, average humidity = 91.7%). The 12 most resistant and most susceptible families over both screens were used to construct the bulks for QTL-seq. All methods were performed in accordance with the institutional and national Biosafety regulations.

### DNA extraction and sequencing

Frozen leaf material from the F_2_ plants of the 24 selected F_2:3_ families was ground using a TissueLyser II (QIAGEN, Hilden, Germany) and DNA was extracted using the E.Z.N.A. HP Plant DNA Mini Kit (Omega Bio-Tek Inc., Norcross, GA). DNA was quantified with an Infinite M200 Pro plate reader (Tecan Group Ltd., Mannedorf, Switzerland) using the i-control software (Tecan Group Ltd.,) and a NanoQuant Plate™ (Tecan Group Ltd.,). Agarose gel electrophoresis was used to confirm the quality of genomic DNA. Equal amounts of DNA from the 12 most resistant and 12 most susceptible families were pooled to create the resistant (R-Bulk) and susceptible bulk (S-Bulk), respectively. The samples were sent to Novogene (Novogene Corporation Inc., Davis, CA) for whole genome sequencing on an Illumina Platform (PE150, Q30 > 80%).

### QTL-seq

The raw reads were combined into forward and reverse read files for each bulk and checked for quality using FastQC^42^. The reads were then aligned to the *C. lanatus* 97103_v2 genome using BWA-MEM^24,43^. The resulting SAM files were converted into BAM files using SAMtools, which was also used to sort the reads by alignment position and then index the resulting BAM files^44^. SAMtools was used to calculate mapping and pairing ratios of raw reads. BEDtools was then used to calculate average read depth for the BAM files^45^. Read group names were standardized using Picard Tools (http://broadinstitute.github.io/picard/), which was then used to mark duplicate reads and index the files. SAMtools was used to index the 97103_v2 genome, and Picard Tools was used to create a dictionary file for the 97103_v2 genome. Reads were re-aligned using GATK to generate clean reads from misaligned regions^46^. GATK was used to perform variant calling and the resulting VCF files from each bulk were combined into a single VCF file. GATK was used to filter the VCF file so that only SNPs remained. These SNPs were then filtered using GATK (QD < 2.0 || FS > 60.0 || MQ < 40.0 || MQRankSum < − 12.5 || ReadPosRankSum < − 8.0), and filtered SNPs were then removed using VCFtools^47^. GATK was used to output the SNPs from the VCF file into a table format suitable for use in the R package QTLseqr^48,49^. After importation into QTLseqr, SNPs were again filtered (refAlleleFreq = 0.2, minTotalDepth = 100, maxTotalDepth = 150, minSampleDepth = 40, minGQ = 99), and then used to calculate the Δ-SNP index at each SNP^18^. This Δ-SNP index was used with a 1 Mb sliding window to calculate a smoothed Δ-SNP index as well as 95% and 99% confidence intervals for a region’s contribution to the trait of interest (GSB resistance).

### Primer design and genotyping

KASP (LGC Genomics LLC, Teddington, UK) primers were designed for SNPs spanning the regions of interest identified by QTLseqr using Primer3Plus and the *C. lanatus* 97103_v2 genome^24^. DNA was extracted for parents, F_1,_ and all F_2_ plants as previously described. KASP PCR reactions contained 1.94 μL of 2 × KASP Master Mix (LGC Genomics LLC), 0.06 μL of KASP Primer Mix, and 2 μL of DNA (10–20 ng/μl) in a total volume of 4 μL. The KASP Primer Mix contained 12 μL of each forward primer (100 μM), 30 μL of reverse primer (100 μM), and 46 μL of sterile distilled water. The following PCR conditions were used: 95 °C for 15 min followed by 10 touchdown cycles of 95 °C for 20 s, 66 °C for 25 s, and 72 °C for 15 s, followed by 35 cycles of 95 °C for 10 s, 57 °C for 60 s and 72 °C for 15 s. KASP fluorescent end readings were measured using an Infinite M200 Pro (Tecan Group Ltd.) plate reader using the Magellan (Tecan Group Ltd.) software. Genotypes were called using KlusterCaller™ (LGC Genomics LLC).

### QTL mapping

A genetic map (n = 111) was created of each region of interest using the following settings in ICIMapping^50^: Grouping was performed by recombination frequency with a threshold value of 0.30, ordering was performed k-Optimally by recombination frequency using the 2-OptMAP algorithm with 10 NN initials, rippling was performed by recombination frequency with a window size of 5. Resulting genetic maps were visualized using MapChart^51^. The genetic map was then used for QTL mapping using ICIMapping with the following settings^50^: The ICIM-ADD mapping method was used with deletion of missing phenotypes, a 1 cM step, and a value of 0.001 for the probability in stepwise regression. The LOD threshold of 2.5079 was determined by running 1000 permutations. The resulting data was graphed using R and the ggplot package^49,52^. Digenic epistais was investigated using the ICIM-EPI mothod in ICIMapping^50^. One-way ANOVAs and Tukey–Kramer tests were performed using JMP® version 15.0.0 (SAS Institute Inc., Cary, NC) to test the association between markers and phenotype, as well as a haplotype representing flanking markers for *Qgsb5.2* simultaneously, and GSB disease severity in the population.

### Synteny analysis and candidate genes

The cucurbit genomics genome browser (http://cucurbitgenomics.org) was used to identify gene predictions for the *C. lanatus* 97103v2 genome^24,53^. These genes were then examined for predicted functions related to disease resistance. The cucurbit genomics synteny browser (http://cucurbitgenomics.org) was used to check the synteny of the *Qgsb5.2* to other reported GSB resistance QTL in cucurbits.

## Data Availability

The sequencing data used during the current study available from NCBI Sequence Read Archive (PRJNA873687).
